# The Impact of Metabolic Syndrome Components on Erectile Function in Patients with Type 2 Diabetes

**DOI:** 10.3390/metabo13050617

**Published:** 2023-04-30

**Authors:** Alexandra Katsimardou, Dimitrios Patoulias, Ioanna Zografou, Fotios Siskos, Konstantinos Stavropoulos, Konstantinos Imprialos, Zoi Tegou, Aristi Boulmpou, Vivian Georgopoulou, Nikoleta Hatzipapa, Christodoulos Papadopoulos, Michael Doumas

**Affiliations:** 1 2nd Propedeutic Department of Internal Medicine, Aristotle University of Thessaloniki, Hippokration General Hospital, 54642 Thessaloniki, Greece; 2 3rd Department of Cardiology, Aristotle University of Thessaloniki, Hippokration General Hospital, 54642 Thessaloniki, Greece; 3 Veterans Affairs Medical Center, George Washington University, Washington, DC 20422, USA

**Keywords:** erectile dysfunction, sexual dysfunction, type 2 diabetes mellitus, metabolic syndrome, hypertension, obesity, triglycerides, HDL

## Abstract

Erectile dysfunction is commonly encountered in diabetic patients and in patients with metabolic syndrome; however, only a few studies have assessed patients with metabolic syndrome and type 2 diabetes mellitus (T2DM) regarding their sexual function. The purpose of this study is to examine the effect of metabolic syndrome and its components on the erectile function of T2DM patients. A cross-sectional study including T2DM patients was conducted from November 2018 until November 2020. Participants were evaluated for the presence of metabolic syndrome and their sexual function was assessed using the International Index of Erectile Function (IIEF) questionnaire. A total of 45 consecutive male patients participated in this study. Metabolic syndrome was diagnosed in 84.4% and erectile dysfunction (ED) in 86.7% of them. Metabolic syndrome was not associated with ED or ED severity. Among metabolic syndrome components, only high-density lipoprotein cholesterol (HDL) was associated with ED [x^2^ (1, n = 45) = 3.894, *p* = 0.048; OR = 5.5 (95% CI: 0.890–33.99)] and with the IIEF erectile function scores (median 23 vs. 18, U = 75, *p* = 0.012). Multiple regression analyses showed that HDL was non-significantly associated with the IIEF erectile function scores. In conclusion, among T2DM patients HDL is associated with ED.

## 1. Introduction

Type 2 diabetes mellitus (T2DM) is a significant cause of morbidity and mortality worldwide [1]. Epidemiological data suggest that diabetes it is the ninth cause of death, with mortality rates increasing from 2007 to 2017 for T2DM, and the sixth cause of disability worldwide and, by 2030, around 10.2% of the world population is estimated to be affected [1,2,3,4]. Apart from the well-established microvascular and macrovascular complications that arise in the presence of T2DM, erectile dysfunction (ED) is a frequently unrecognized and underreported complication that is commonly encountered in this patient population [5]. It is defined as the persistent or recurrent inability to attain and/or maintain a penile erection sufficient for successful sexual intercourse for at least three months [6]. In particular, diabetic patients have a three-fold higher prevalence of ED, while their symptoms are more severe and more difficult to control with any given medication [7,8]. On the other hand, significant evidence supports that ED can be considered a cardiovascular risk factor. Specifically, based on the artery-size hypothesis, its presence may precede the emergence of clinically evident cardiovascular disease by three to five years, thereby offering patients and clinicians an important therapeutic window to intervene [9,10]. Especially in diabetic men, ED independently predicts coronary artery disease in patients without overt cardiovascular disease, highlighting the importance of ED screening in T2DM patients [11].

Metabolic syndrome, previously known as syndrome X, refers to the simultaneous presence of different combinations of metabolic abnormalities. Among the metabolic derangements included in the definition of metabolic syndrome are hypertension, central obesity, dyslipidemia with elevated triglycerides (TG) and/or reduced high-density lipoprotein cholesterol levels (HDL) and glucose intolerance [12]. Around 35% of the US population is estimated to have metabolic syndrome and, as obesity has emerged as a global epidemic similar to T2DM, more and more patients are expected to meet the criteria for the diagnosis of metabolic syndrome [13,14,15]. Metabolic syndrome is a well-recognized risk factor for cardiovascular disease, in particular, a 2-fold increase in the risk for cardiovascular disease and a 1.5-fold increase for all-cause mortality have been observed in previous studies [16,17]. This observation also stands for the components of the metabolic syndrome, namely hypertension, central obesity, abnormal triglyceride and high-density lipoprotein levels and abnormal glucose metabolism [18,19,20]. Regarding male sexual function, evidence suggests that the presence of metabolic syndrome can be considered a risk factor for the development of ED [21]. In particular, metabolic syndrome is associated with a 2.5-fold increase in the odds of self-reported ED [22].

Only a few studies have assessed the effect of metabolic syndrome on ED in T2DM patients [23,24,25]. Therefore, in the present study, we sought to determine the effect of metabolic syndrome and its components on the erectile function of T2DM patients. Apart from erectile function, all other aspects of male sexual function were examined, based on the International Index of Erectile Function (IIEF) Questionnaire results.

## 2. Materials and Methods

A cross-sectional study, the Diabetic Complications and Erectile Dysfunction study (DIACOMED), was designed and conducted in accordance with the principles of the Helsinki declaration. The study was approved by the Bioethics Committee of the Aristotle University of Thessaloniki and all subjects gave informed consent prior to their enrollment in the study (protocol number: 1649; date of approval: 21 November 2018). The current report constitutes a subanalysis of the DIACOMED study. Consecutive male subjects who visited the outpatient clinic of the Second Propedeutic Department of Internal Medicine of the Aristotle University of Thessaloniki from November 2018 until November 2020 were included. Inclusion criteria were a prior diagnosis of T2DM and age above 18 years, while individuals with an inability or unwillingness to give consent, those with a history of alcohol or substance abuse and those with an acute illness were excluded from the study.

A meticulous medical history was obtained and data regarding age, race, ethnicity, marital status, tobacco and alcohol use, weight, height, and waist circumference measured above the iliac crest at the umbilicus level were collected. Subjects were evaluated for the presence of comorbidities, such as hypertension, dyslipidemia, cardiovascular disease (coronary artery disease, stroke, peripheral artery disease, heart failure), chronic kidney disease and chronic obstructive pulmonary disease. Medication history was also documented and recorded, with a specific emphasis on antidiabetic and antihypertensive medications. Body mass index (BMI) was calculated using the formula weight to the square of height (kg/m^2^).

Laboratory measurements included fasting plasma glucose (FPG), glycated hemoglobin (HbA1c), liver function tests, kidney function tests, HDL, total cholesterol (TCHOL) and TG. All samples were collected in the morning after an 8 h fasting period. The glomerular filtration rate (GFR) was calculated using the Chronic Kidney Disease Epidemiology Collaboration (CKD-EPI) equation [26]. Finally, a 24 h urine collection was performed to assess the presence and stage of albuminuria. Office blood pressure was measured following the European Society of Hypertension guidelines, with the subjects in the sitting position using an automated oscillometric device and the average of the last two out of three readings was used [27].

Metabolic syndrome was diagnosed based on the Joint Interim Societies (JIS) definition that was introduced in 2009. According to that, metabolic syndrome is present when 3 out of 5 of the following criteria are met: central obesity, based on population and country-specific cut-off values, FPG ≥ 100 mg/dL or a history of T2DM, TG ≥ 150 mg/dL or a history of drug treatment, HDL < 40 mg/dL in males and <50 mg/dL in females or a history of specific treatment and finally systolic blood pressure (SBP) ≥ 130 mmHg and/or diastolic blood pressure (DBP) ≥ 85 mmHg or a history of antihypertensive treatment. As for central obesity, the International Diabetes Federation (IDF) cut-off points for Europids were used, which are ≥94 cm for men and ≥80 cm for women [12]. Subjects with metabolic syndrome were subdivided into three categories based on the number of metabolic syndrome criteria met (three out of five, four out of five and five out of five metabolic syndrome criteria met).

For the assessment of sexual function, the IIEF was used. Specifically, the IIEF is a 15-item questionnaire that refers to five domains of male sexual function, namely erectile function, orgasmic function, sexual desire, intercourse satisfaction and overall satisfaction [28]. A score below 25 for erectile function is indicative of the presence of erectile dysfunction, while patients may be further classified into five categories according to the ED severity: mild ED, for erectile function scores from 22 to 25; mild to moderate ED, for erectile function scores from 17 to 21; moderate ED, for erectile function scores from 11 to 16; and finally severe ED, for erectile function scores from 6 to 10 [29].

Subgroup analysis was also performed. Patients with metabolic syndrome were divided into two distinct groups, those who had a higher metabolic burden and met all the criteria and those who met three and four out of five criteria for the diagnosis of metabolic syndrome. Similarly, those with ED were divided into two categories based on ED severity; those with mild and mild to moderate ED (erectile function score 17–25) and those with moderate and severe ED (erectile function scores < 17).

Statistical analysis was performed with the use of the Statistical Package for Social Sciences (IBM SPSS Statistics, 28.0 version). Data analysis was performed at the 0.05 level of significance. Qualitative variables were described as frequencies/percentages and the χ^2^ test was used to assess any differences among them. Quantitative variables were assessed for normality using the Shapiro–Wilk test. Data for variables with a normal distribution were presented as mean ± standard deviation (SD) and for those with an abnormal distribution as median and 25–75% percentiles. Based on the normality test, parametric or non-parametric tests were further implemented. Specifically, Student’s *t*-test, ANOVA and Pearson’s correlation analysis were used for those variables with a normal distribution, while Mann–Whitney, Kruskal–Wallis and Spearman’s correlation analysis were used for variables with an abnormal distribution. For ED and ED categories, univariate and multivariate logistic regression analysis was performed. Variables included in the equation were chosen based on the beforementioned statistical analyses or their proven contribution to ED from previously published studies. Similarly, univariate and multiple linear regression analysis was performed separately for the five domains of sexual function of the IIEF questionnaire.

## 3. Results

From November 2018 until November 2020, 45 consecutive male subjects with T2DM gave informed consent to participate in our study. Among them, 15.6% were single, 2.2% were in a relationship, 73.3% were married, 6.7% were divorced and 2.2% were widowed. The mean age of the participants was 62.8 (SD 10.54) years, the median duration of T2DM was 10 (4–15) years and the median HbA1c was 7.3% (6.5–8.05), as depicted in Table 1. Therefore, 71.1% of the participants did not meet the glycemic goal of an HbA1c below 7%, as suggested by current guidelines. Moreover, 44.4%, 22.2%, 22.2% and 40% were diagnosed with diabetic kidney disease, diabetic retinopathy, diabetic peripheral neuropathy and cardiac autonomic neuropathy (CAN), respectively.

As the study enrolled T2DM patients, all of them were on antiglycemic medication. In particular, 86.7%, 13.3%, 17.8%, 6.7%, 24.4 % and 28.9% of them were on metformin, glucagon-like peptide-1 receptor agonist, sodium-glucose cotransporter-2 inhibitor, sulfonylurea, dipeptidyl peptidase-4 inhibitor and insulin treatment, respectively. No differences were observed among those with or without metabolic syndrome regarding hypoglycemic medication. More patients with metabolic syndrome received a renin–angiotensin–aldosterone system (RAAS) inhibitor or a statin than those without metabolic syndrome (for RAAS inhibitors: 63.2% vs. 14.3%, *p* = 0.017; for statin: 63.2% vs. 0%, *p* = 0.002). Furthermore, 35.6% received a diuretic and 46.7% were on treatment with a b blocker; further details regarding medication are depicted in more detail in Supplementary Table S1.

Metabolic syndrome was diagnosed in 84.4% of the study population. As patients were already diagnosed with T2DM, the presence of two more out of four diagnostic criteria was required for the diagnosis. Specifically, 75.6% had an elevated waist circumference, 77.8% were on antihypertensive treatment or had systolic and/or diastolic blood pressure above the limit of 130 and/or 80 mmHg, 80% were on hypolipidemic treatment or had an HDL below 40 mg/dL and finally, 77.8% were on treatment or had triglycerides above 150 mg/dL. Among patients with metabolic syndrome, 7.9% fulfilled three out of five criteria, 28.9% fulfilled four out of five criteria and most of them, namely 63.2%, met all the criteria for the diagnosis of metabolic syndrome.

ED was diagnosed in 86.7% of the participants. In particular, 20%, 26.7%, 8.9% and 31.1% suffered from mild, mild to moderate, moderate and severe ED, respectively, while the median erectile function score was 18 (9–22) (Table 2). Among those with ED, 87.2% (85.7% with mild and mild to moderate ED and 88.9% with moderate and severe ED (*p* = 0.768)) were diagnosed with metabolic syndrome. Metabolic syndrome was not associated with the presence nor with the severity of ED (x^2^ (1, n = 45) = 1.666, *p* = 0.197; x^2^ (1, n = 45) = 3.251, *p* = 0.517, respectively). Furthermore, no significant difference was documented among those with and without metabolic syndrome concerning the five domains of sexual function, as described in Table 2. Interestingly, among patients with metabolic syndrome and ED, the majority of them, namely 67.6%, met all the criteria compared to 32.4% who met three or four out of five criteria for metabolic syndrome diagnosis (x^2^ (1, n = 38) = 6.855, *p* = 0.009). However, multiple logistic regression analysis failed to verify this association after age and diabetes duration were taken into account.

Among different components of metabolic syndrome, abnormal HDL was significantly associated with the presence of ED (x^2^ (1, n = 45) = 3.894, *p* = 0.048; OR = 5.5 (95% CI: 0.890–33.99)) and with the IIEF erectile function (IIEF-EF) scores (median 23 vs 18, U = 75, *p* = 0.012), whereas hypertension, central obesity and abnormal triglycerides were not (Table 3).

As for ED, univariate and multiple logistic regression analysis failed to show any significant association for metabolic syndrome and its components, including HDL. On the contrary, for IIEF-EF scores, univariate linear regression analysis revealed that age and HDL were significant factors. Since the study population differed by age, diabetes duration and the presence of diabetic complications, multiple linear regression analysis was performed. Regarding diabetic complications, diabetic retinopathy and cardiac autonomic neuropathy were excluded from the analysis, as they were both found to be significantly correlated with diabetes duration (r = 0.561, *p* = 0.001 for diabetic retinopathy and r = 0.326, *p* = 0.033 for cardiac autonomic neuropathy, respectively). When patients were adjusted for age, diabetes duration and diabetic complications, such as for the presence of diabetic nephropathy and diabetic peripheral neuropathy, only the presence of an abnormal HDL exerted an effect on the IIEF-EF score, although the effect was non-significant (R^2^ = 0.222, F(5, 39) = 2.22, *p* = 0.072) (Table 4).

Among patients with ED, worse glycemic control was associated with worse erectile function scores. Specifically, those with mild and mild to moderate ED had lower median HbA1c levels and lower median FPG compared to those with moderate and severe ED (for HbA1c: median 7% versus 7.55%, U = 269, *p* = 0.024; for FPG: median 129 mg/dL vs. 186 mg/dL, U = 254.5, *p* = 0.024, accordingly). Furthermore, multiple linear regression analysis was used to examine the associations between age, diabetes duration and HbA1c and IIEF-EF scores. Two predictors, age and HbA1c, explained 21.7% of the variance (R^2^ = 0.217, F (3, 41) = 3.782, *p* = 0.017). Specifically, for every 1% increase in HbA1c, IIEF-EF scores were expected to drop by 2.11 points (β = −2.112, *p* = 0.02), while for every increase in age by 10 years, IIEF-EF scores were expected to drop by approximately three points (β = −0.306, *p* = 0.007) (Table 5). No association was documented between the other aspects of sexual function (orgasmic function, sexual desire, intercourse satisfaction and overall satisfaction) and the indices of glycemic control.

## 4. Discussion

In this cross-sectional study, where ED was present in 86.7% of the study population, the presence of metabolic syndrome was not associated with ED as an entity or ED severity. Among the components of metabolic syndrome, however, abnormal HDL was associated with ED and IIEF-EF scores. Moreover, age and HDL were proven to be significant predictors of the IIEF-EF score in univariate regression analyses. However, multiple regression analyses showed that only HDL remains a determinant for the IIEF-EF score when age, diabetes duration and the presence of diabetic complications, such as nephropathy or diabetic peripheral neuropathy, are taken into consideration, although this association is non-significant.

Erectile function is a complex process involving psychological, vascular, neurological and endocrine mechanisms. It is mostly dependent on the nitric oxide (NO) bioavailability in the smooth muscle cells of the corpus cavernosum. Erectile dysfunction affects males progressively as they age. In the European Male Ageing Study, the prevalence of moderate or severe ED was 19%, 38% and 64% among participants that were 50–59, 60–69 and above 70 years of age, respectively [30]. In our study, the mean age of the participants was 62.8 (SD 10.54) years and, in agreement with the beforementioned results, age contributed significantly to the presence of lower IIEF-EF scores. Apart from age, the presence of diabetes mellitus, hypertension, dyslipidemia, depression, smoking, obesity, physical inactivity and several other factors seem to contribute to the emergence of ED [31].

In our study involving T2DM patients, ED was diagnosed in 86.7% of the participants. Results from a recent meta-analysis revealed that ED affects two-thirds of T2DM patients, while even those with a short duration of T2DM may be affected, as was evident from another study that included males below 45 years of age [25,32]. In the latter study, the presence of T2DM was associated with a worse IIEF-5 score and a 3.5 increased odds ratio for having moderate to severe ED. Interestingly, T2DM patients had a short duration of diabetes (mean duration 2.8 years) and poor glycemic control (mean HbA1c 9.1%). In the same study, no differences were reported among T2DM patients with or without metabolic syndrome regarding ED [25].

The presence of T2DM is associated with more severe ED; specifically, an 8.4-fold increased relative risk for severe ED (*p* < 0.001, 95% CI 2.8–24.6) has been documented [23]. Furthermore, our data suggest that worse glycemic control is associated with worse erectile function. Indeed, higher HbA1c levels have been correlated with ED in previous reports [33]. Poor glycemic control has been associated with worse clinical outcomes and the emergence of many diabetic complications [34,35]. Through distinct pathophysiologic mechanisms, microangiopathy and macroangiopathy may emerge, situations that are closely related to the emergence of ED in diabetic patients alongside autonomic neuropathy [36,37,38]. Therefore, it could be hypothesized that efforts to optimize glycemic control, beyond other well-established benefits, could also contribute to the prevention of the emergence and progression of ED in these patients, although such an association has not been clearly established in T2DM patients.

Metabolic syndrome was not associated with ED, while among dyslipidemia, hypertension and central obesity, only abnormal HDL was significantly associated with the presence of ED. Our results differ from previous reports, like those of a prospective study of male T2DM patients that sought to determine the relationship between the presence of metabolic syndrome and its components with erectile dysfunction, where 84.6% had metabolic syndrome and 90.9% suffered from ED based on their IIEF-5 scores. Age, duration of T2DM, blood pressure and the level of triglycerides were significantly associated with the presence of ED, whereas the presence of metabolic syndrome, HDL-c levels, BMI and the duration of hypertension were not [24]. In this study, only erectile function was assessed while in our study all aspects of male sexual function were evaluated. Similarly, in another study between Chinese T2DM patients ED was diagnosed in 84.3% of them based on the IIEF-5 questionnaire, while among waist circumference, HDL, TG and hypertension, only the latter was associated with the presence of ED [39]. In contrast, impaired lipid metabolism was associated with ED in a previous study that involved diabetic and non-diabetic patients with ED. HDL was significantly associated with ED, while a significant decrease in five apolipoproteins (H, A4, J, E, A1) was observed that was also associated with older age. Apart from the reduced levels of those apolipoproteins, oxidative changes were also proven, further enhancing the role of impaired lipid metabolism in ED [40]. Moreover, our results show that among patients with metabolic syndrome, those who met all the criteria and had a higher burden of disease were more likely to suffer from ED compared to those who met three or four criteria. Similar results were documented by a previous study that was not limited to T2DM patients, in which the number of metabolic risk factors was inversely correlated with the IIEF erectile function scores [21].

Specifically for HDL-cholesterol, however, higher levels have been associated with better erection and low levels have been associated with arteriogenic ED in previous reports [41,42]. HDL participates in reverse cholesterol transport by removing cholesterol from peripheral tissues and delivering it towards the liver and other steroidogenic organs [43]. However, apart from its role as a “scavenger”, HDL has a significant role as an anti-inflammatory, antithrombotic and antiapoptotic factor. In detail, it has a protective role towards the endothelium, as it seems that HDL attenuates apoptosis, stimulates proliferation and exerts anti-inflammatory actions towards endothelial cells, it attenuates degradative and proinflammatory processes on vascular smooth muscle cells and, last but not least, HDL enhances the activity of endothelial nitric oxide (eNOS) synthase [44,45,46,47,48]. Impaired eNOS activity and reduced NO levels that subsequently lead to endothelial dysfunction and reduced smooth muscle and vascular relaxation are characteristic for ED [49]. Moreover, diabetes mellitus exerts qualitative, quantitative, and kinetic abnormalities on lipoproteins. Specifically for HDL, increased catabolism, reduced levels and increased TG content and glycation of apolipoproteins have been described [50]. Collectively, as HDL is protective for the vasculature, it could also be protective for erectile function and our study results clearly demonstrate that there is an association among them.

Our study has certain limitations. The small sample size of the study population limits the generalizability of our results. Furthermore, since our study was a cross-sectional study, causality cannot be established. Further large-scale prospective studies are needed to unveil the pathophysiologic mechanisms that are implicated in the association between ED and HDL if any are present.

## 5. Conclusions

In conclusion, ED and metabolic syndrome are commonly encountered in T2DM patients, as was also evident in our study. HDL was significantly associated with the presence and the severity of ED; however, more evidence from prospective randomized controlled studies is needed to establish the exact association among them, as well as to clarify whether treatment of HDL may lead to better IIEF erectile function scores.

## Figures and Tables

**Table 1:** Main demographic and clinical characteristics of the study population.

	Total Patients *N* = 45	With Metabolic Syndrome *N* = 38	Without Metabolic Syndrome *N* = 7	*p*
Age (y) *	62.8 (10.54)	62.71 (10.14)	63.29 (13.45)	0.896
*Diabetes*				
Diabetes duration (y) **	10 (4–15)	9.5 (3–15)	10 (7–12)	0.842
HbA1c (%) **	7.3 (6.5–8.05)	7.35 (6.75–8.12)	7 (6.2–7.5)	0.187
HbA1c below 7%	13 (28.9%)	10 (26.3%)	3 (42.9%)	0.375
FPG (mg/dL) **	146 (122–194)	172 (126.75–198.75)	116 (89–129)	0.002
*Dyslipidemia*				
Abnormal TG or treatment	35 (77.8%)	35 (92.1%)	0 (0%)	0.001
TG (mg/dL) **	137 (86.5–245)	151 (91.5–265.5)	102 (55.25–113.5)	0.041
Abnormal HDL or treatment	36 (80%)	35 (92.1%)	1 (14.3%)	0.001
HDL (mg/dL) **	38 (34–44)	36.5 (33–43)	44.5 (39.25–57.5)	0.033
*Obesity*				
Central obesity	34 (75.6 %)	32 (84.2%)	2 (28.6%)	0.002
BMI (kg/m^2^) *	29.58 (4.56)	29.36 (5.03)	25.5 (7.64)	0.035
WC (cm) *	102.69 (10.66)	104.11 (9.56)	95 (13.72)	0.018
Hypertension	35 (77.8%)	33 (86.8%)	2 (28.6%)	0.001
Hypertension duration (y) **	8 (3–15)	8 (4–15)	6.5 (1–12)	0.498
*ED*	39 (86.7%)	34 (89.5%)	5 (71.4%)	0.197

Expressed as n (%), mean (SD) *, median (25th–75th percentile) **. BMI: body mass index; ED: erectile dysfunction; FPG: fasting plasma glucose; HbA1c: glycated hemoglobin; HDL: high-density lipoprotein; IIEF: International Index of Erectile Function Score; WC: waist circumference.

**Table 2:** IIEF scores among patients with and without MetS.

	Total Patients *N* = 45	With Metabolic Syndrome *N* = 38	Without Metabolic Syndrome *N* = 7	*p*
Erectile function *	18 (9–22)	18 (9–22)	23 (13–28)	0.157
Orgasmic function *	8 (5–9)	8 (4–9)	7 (5–9)	0.748
Sexual desire *	6 (4–9)	6 (4–9)	7 (6–9)	0.570
Intercourse satisfaction	8.42 (3.27)	8.18 (3.23)	9.71 (3.45)	0.260
Overall satisfaction	5.84 (2.35)	5.79 (2.34)	6.14 (2.61)	0.720

Expressed as mean (SD), median (25th–75th percentile) *. IIEF: International Index of Erectile Function Score; MetS: metabolic syndrome.

**Table 3:** Associations among ED and MetS and its components.

	With ED *N* = 39	Without ED *N* = 6	*p*
Hypertension	32 (82.1%)	3 (50%)	0.079
Central obesity	31 (79.5%)	3 (50%)	0.118
Abnormal HDL	33 (84.6%)	3 (50%)	0.048
Abnormal TG	31 (79.5%)	4 (66.7%)	0.482
Metabolic syndrome	34 (87.2%)	4 (66.7%)	0.197

Expressed as n (%). ED: erectile dysfunction; HDL: high-density lipoprotein; TG: triglycerides.

**Table 4:** The associations among age and HDL and the IIEF erectile function score.

Model	Unstandardized Coefficients		Standardized Coefficients	t	Sig.	95.0% CI for B	
	B	Std. Error	Beta			Lower Bound	Upper Bound
(Constant)	34.700	6.613		5.248	0.000	21.325	48.075
Age	−0.208	0.108	−0.302	−1.918	0.062	−0.426	0.011
Diabetes duration	0.073	0.141	0.085	0.517	0.608	−0.213	0.359
Diabetic nephropathy	−0.225	2.181	−0.016	−0.103	0.918	−4.638	4.187
Diabetic peripheral neuropathy	−2.229	2.502	−0.129	−0.891	0.378	−7.290	2.831
HDL < 40 mg/dL or specific treatment	−6.132	2.657	−0.342	−2.308	0.026	−11.507	−0.758

CI: confidence interval; HDL: high-density lipoprotein.

**Table 5:** Associations among age, diabetes duration and HbA1c and IIEF erectile function score.

Model	Unstandardized Coefficients		Standardized Coefficients	t	Sig.	95.0% CI for B	
	B	Std. Error	Beta			Lower Bound	Upper Bound
(Constant)	49.870	9.968		5.003	0.000	29.741	70.000
Age	−0.306	0.108	−0.445	−2.846	0.007	−0.523	−0.089
Diabetes duration	0.190	0.133	0.221	1.426	0.161	−0.079	0.458
HbA1c	−2.112	0.874	−0.345	−2.415	0.020	−3.878	−0.346

CI: confidence interval; HbA1c: glycated hemoglobin.

## Data Availability

Data may be available on request due to privacy restrictions.

## Supplementary Materials

The following supporting information can be downloaded at: https://www.mdpi.com/article/10.3390/metabo13050617/s1, Table S1: Medication of the study’s population.

## References

[1] Khan M.A.B., Hashim M.J., King J.K., Govender R.D., Mustafa H., Al Kaabi J. (2020). Epidemiology of type 2 diabetes—Global burden of disease and forecasted trends. J. Epidemiol. Glob. Health.

[2] GBD 2015 Disease and Injury Incidence and Prevalence Collaborators (2016). Global, regional, and national incidence, prevalence, and years lived with disability for 310 diseases and injuries, 1990–2015: A systematic analysis for the Global Burden of Disease Study 2015. Lancet.

[3] Saeedi P., Petersohn I., Salpea P., Malanda B., Karuranga S., Unwin N., Colagiuri S., Guariguata L., Motala A.A., Ogurtsova K. (2019). Global and regional diabetes prevalence estimates for 2019 and projections for 2030 and 2045: Results from the International Diabetes Federation Diabetes Atlas, 9th edition. Diabetes Res. Clin. Pract..

[4] Roth G.A., Abate D., Abate K.H., Abay S.M., Abbafati C., Abbasi N., Abbastabar H., Abd-Allah F., Abdela J., Abdelalim A. (2018). Global, regional, and national age-sex-specific mortality for 282 causes of death in 195 countries and territories, 1980–2017: A systematic analysis for the Global Burden of Disease Study 2017. Lancet.

[5] Malavige L.S., Levy J.C. (2009). Erectile dysfunction in diabetes mellitus. J. Sex. Med..

[6] NIH Consensus Development Panel on Impotence (1993). Impotence—NIH Consensus Conference. JAMA.

[7] Lewis R.W. (2001). Epidemiology of erectile dysfunction. Urol. Clin. N. Am..

[8] Hackett G., Kirby M., Wylie K., Heald A., Ossei-Gerning N., Edwards D., Muneer A. (2018). British society for sexual medicine guidelines on the management of erectile dysfunction in men—2017. J. Sex. Med..

[9] Thompson I.M., Tangen C.M., Goodman P.J., Probstfield J.L., Moinpour C.M., Coltman C.A. (2005). Erectile dysfunction and subsequent cardiovascular disease. JAMA.

[10] Viigimaa M., Vlachopoulos C., Doumas M., Wolf J., Imprialos K., Terentes-Printzios D., Ioakeimidis N., Kotsar A., Kiitam U., Stavropoulos K. (2020). Update of the position paper on arterial hypertension and erectile dysfunction. J. Hypertens..

[11] Ma R.C.-W., So W.-Y., Yang X., Yu L.W.-L., Kong A.P.-S., Ko G.T.-C., Chow C.-C., Cockram C.S., Chan J.C.-N., Tong P.C.-Y. (2008). Erectile Dysfunction Predicts Coronary Heart Disease in Type 2 Diabetes. J. Am. Coll. Cardiol..

[12] Alberti K.G.M.M., Eckel R.H., Grundy S.M., Zimmet P.Z., Cleeman J.I., Donato K.A., Fruchart J.C., James W.P.T.J., Loria C.M., Smith S.C. (2009). Harmonizing the metabolic syndrome: A joint interim statement of the international diabetes federation task force on epidemiology and prevention; National heart, lung, and blood institute; American heart association; World heart federation; International. Circulation.

[13] Aguilar M., Bhuket T., Torres S., Liu B., Wong R.J. (2015). Prevalence of the metabolic syndrome in the United States, 2003–2012. JAMA.

[14] Shaw J.E., Sicree R.A., Zimmet P.Z. (2010). Global estimates of the prevalence of diabetes for 2010 and 2030. Diabetes Res. Clin. Pract..

[15] Ward Z.J., Bleich S.N., Cradock A.L., Barrett J.L., Giles C.M., Flax C., Long M.W., Gortmaker S.L. (2019). Projected U.S. State-level prevalence of adult obesity and severe obesity. N. Engl. J. Med..

[16] Mottillo S., Filion K.B., Genest J., Joseph L., Pilote L., Poirier P., Rinfret S., Schiffrin E.L., Eisenberg M.J. (2010). The metabolic syndrome and cardiovascular risk: A systematic review and meta-analysis. J. Am. Coll. Cardiol..

[17] Athyros V., Ganotakis E., Kolovou G.D., Nicolaou V., Achimastos A., Bilianou E., Alexandrides T., Karagiannis A., Paletas K., Liberopoulos E. (2011). Assessing the treatment effect in metabolic syndrome without perceptible diabetes (ATTEMPT): A prospective-randomized study in middle aged men and women. Curr. Vasc. Pharm..

[18] Galassi A., Reynolds K., He J. (2006). Metabolic Syndrome and risk of cardiovascular disease: A meta-analysis. Am. J. Med..

[19] Berry J.D., Dyer A., Cai X., Garside D.B., Ning H., Thomas A., Greenland P., Van Horn L., Tracy R.P., Lloyd-Jones D.M. (2012). Lifetime risks of cardiovascular disease. N. Engl. J. Med..

[20] Joseph P., Leong D., McKee M., Anand S.S., Schwalm J.-D., Teo K., Mente A., Yusuf S. (2017). Reducing the global burden of cardiovascular disease, part 1. Circ. Res..

[21] Demir T., Demir O., Kefi A., Comlekci A., Yesil S., Esen A. (2006). Prevalence of erectile dysfunction in patients with metabolic syndrome. Int. J. Urol..

[22] Weinberg A.E., Eisenberg M., Patel C.J., Chertow G.M., Leppert J.T. (2013). Diabetes severity, metabolic syndrome, and the risk of erectile dysfunction. J. Sex. Med..

[23] Aslan Y., Sezgin T., Tuncel A., Tekdogan U.Y., Guler S., Atan A. (2009). Is type 2 diabetes mellitus a cause of severe erectile dysfunction in patients with metabolic syndrome?. Urology.

[24] Chaudhary R.K., Shamsi B.H., Tan T., Chen H.-M., Xing J.-P. (2016). Study of the relationship between male erectile dysfunction and type 2 diabetes mellitus/metabolic syndrome and its components. J. Int. Med. Res..

[25] Wang C.C., Chancellor M.B., Lin J.M., Hsieh J.H., Yu H.J. (2010). Type 2 diabetes but not metabolic syndrome is associated with an increased risk of lower urinary tract symptoms and erectile dysfunction in men aged <45 years. BJU Int..

[26] Levey A.S., Stevens L.A., Schmid C.H., Zhang Y.L., Castro A.F., Feldman H.I., Kusek J.W., Eggers P., Van Lente F., Greene T. (2009). A New Equation to Estimate Glomerular Filtration Rate. Ann. Intern. Med..

[27] Stergiou G.S., Palatini P., Parati G., O’brien E., Januszewicz A., Lurbe E., Persu A., Mancia G., Kreutz R. (2021). 2021 European Society of Hypertension practice guidelines for office and out-of-office blood pressure measurement. J. Hypertens..

[28] Rosen R.C., Riley A., Wagner G., Osterloh I.H., Kirkpatrick J., Mishta A. (1997). The international index of erectile function (IIEF): A multidimensional scale for assessment of erectile dysfunction. Urology.

[29] Cappelleri J.C., Rosen R.C., Smith M.D., Mishra A., Osterloh I.H. (1999). Diagnostic evaluation of the erectile function domain of the International Index of Erectile Function. Urology.

[30] Corona G., Lee D.M., Forti G., O’Connor D.B., Maggi M., O’Neill T.W., Pendleton N., Bartfai G., Boonen S., Casanueva F.F. (2010). Age-related changes in general and sexual health in middle-aged and older men: Results from the European Male Ageing Study (EMAS). J. Sex. Med..

[31] Shamloul R., Ghanem H. (2013). Erectile dysfunction. Lancet.

[32] Kouidrat Y., Pizzol D., Cosco T., Thompson T., Carnaghi M., Bertoldo A., Solmi M., Stubbs B., Veronese N. (2017). High prevalence of erectile dysfunction in diabetes: A systematic review and meta-analysis of 145 studies. Diabet. Med..

[33] Giugliano F., Maiorino M., Bellastella G., Gicchino M., Esposito K. (2010). Determinants of erectile dysfunction in type 2 diabetes. Int. J. Impot. Res..

[34] Barrett E.J., Liu Z., Khamaisi M., King G.L., Klein R., Klein B.E.K., Hughes T.M., Craft S., Freedman B.I., Bowden D.W. (2017). Diabetic microvascular disease: An endocrine society scientific statement. J. Clin. Endocrinol. Metab..

[35] UK Prospective Diabetes Study (UKPDS) Group (1998). Intensive blood-glucose control with sulphonylureas or insulin compared with conventional treatment and risk of complications in patients with type 2 diabetes (UKPDS 33). Lancet.

[36] Russell NDF, Cooper ME (2015). 50 years forward: Mechanisms of hyperglycaemia-driven diabetic complications. Diabetologia.

[37] Chatterjee S., Khunti K., Davies M.J. (2017). Type 2 diabetes. Lancet.

[38] Faselis C., Katsimardou A., Imprialos K., Deligkaris P., Kallistratos M.S., Dimitriadis K. (2020). Microvascular complications of type 2 diabetes mellitus. Curr. Vasc. Pharm..

[39] Yu L.W., Kong A.P., Tong P.C., Tam C., Ko G.T., Ho C.-S., So W.-Y., Ma R.C., Chow C.-C., Chan J. (2010). Evaluation of erectile dysfunction and associated cardiovascular risk using structured questionnaires in Chinese type 2 diabetic men. Int. J. Androl..

[40] Belba A., Cortelazzo A., Andrea G., Durante J., Nigi L., Dotta F., Timperio A.M., Zolla L., Leoncini R., Guerranti R. (2016). Erectile dysfunction and diabetes: Association with the impairment of lipid metabolism and oxidative stress. Clin. Biochem..

[41] Xu Z., Xu H., Jiang S., Xu Q., Ding K., Zhang D., Guan Y., Zhao S. (2021). Effect of high-density lipoprotein on penile erection: A cross-sectional study. Andrologia.

[42] Liu G., Zhang Y., Zhang W., Wu X., Huang H., Jiang H., Zhang X. (2022). Significance of detailed hematological parameters as markers of arteriogenic erectile dysfunction. Andrology.

[43] Connelly M.A., Williams D.L. (2004). Scavenger receptor BI: A scavenger receptor with a mission to transport high density lipoprotein lipids. Curr. Opin. Lipidol..

[44] Suc I., Escargueil-Blanc I., Troly M., Salvayre R., Nègre-Salvayre A. (1997). HDL and ApoA prevent cell death of endothelial cells induced by oxidized LDL. Arterioscler. Thromb. Vasc. Biol..

[45] Sugano M., Tsuchida K., Makino N. (2000). High-density lipoproteins protect endothelial cells from tumor necrosis factor-alpha-induced apoptosis. Biochem. Biophys. Res. Commun..

[46] Zhang Q., Yin H., Liu P., Zhang H., She M. (2010). Essential role of HDL on endothelial progenitor cell proliferation with PI3K/Akt/cyclin D1 as the signal pathway. Exp. Biol. Med. (Maywood).

[47] Mineo C., Shaul P.W. (2012). Novel biological functions of high-density lipoprotein cholesterol. Circ. Res..

[48] Mineo C., Deguchi H., Griffin J.H., Shaul P.W. (2006). Endothelial and antithrombotic actions of HDL. Circ. Res..

[49] Castela Â., Costa C. (2016). Molecular mechanisms associated with diabetic endothelial–erectile dysfunction. Nat. Rev. Urol..

[50] Athyros V.G., Doumas M., Imprialos K.P., Stavropoulos K., Georgianou E., Katsimardou A., Karagiannis A. (2018). Diabetes and lipid metabolism. Hormones.

