# Supplementary material for: The Impact of Metabolic Syndrome Components on Erectile Function in Patients with Type 2 Diabetes
Source: Metabolites. 2023 Apr 30;13(5):617. doi: 10.3390/metabo13050617 (PMC10222854; doi:10.3390/metabo13050617)
Supplement: Supplementary file 1 [file metabolites-13-00617-s001.zip › metabolites-2251560-supplementary.pdf]

Supplementary Table S1. Medication of the study population.

	Total patients *N*=45	With metabolic syndrome *N*=38	Without metabolic syndrome *N*=7	*p*
Metformin	39 (86.7%)	34 (89.5%)	5 (71.4%)	0.197
GLP1-RA	6 (13.3%)	5 (13.2%)	1 (14.3%)	0.936
SGLT2i	8 (17.8%)	7 (18.4%)	1 (14.3%)	0.793
Sulfonyluria	3 (6.7%)	3 (7.9%)	0 (0%)	0.442
DPP-4 inhibitors	11 (24.4%)	9 (23.7%)	2 (28.6%)	0.782
Insulin	13 (28.9%)	9 (23.7%)	4 (57.1%)	0.073
ACEi/ARB	25 (55.6%)	24 (63.2%)	1 (14.3%)	0.017
CCB	17 (37.8%)	15 (39.5%)	2 (28.6%)	0.585
Diuretic (thiazide or loop)	16 (35.6%)	13 (34.2%)	3 (42.9%)	0.661
B blocker	21 (46.7%)	19 (50%)	2 (28.6%)	0.296
MRA	3 (6.7%)	2 (5.3%)	1 (14.3%)	0.379
Statin	24 (53.3%)	24 (63.2%)	0 (0%)	0.002

Expressed as n(%)

ACEi: Angiotensin-converting enzyme inhibitors; ARB: Angiotensin receptor blockers; CCB: Calcium channel blocker; DPP-4: Dipeptidyl peptidase-4; GLP1-RA: Glucagon-like peptide-1 receptor agonist; MRA: Mineralocorticoid receptor antagonist; SGLT2i: Sodium-glucose Cotransporter-2 inhibitor
